# A retrospective multicentre study on the outcomes of the pharmacist-managed Diabetes Medication Therapy Adherence Clinic programme in primary health clinics across Johor, Malaysia

**DOI:** 10.51866/oa.767

**Published:** 2025-06-24

**Authors:** Chia Woon Tai

**Affiliations:** 1 Pharmacy Unit, Mahmoodiah Health Clinic Johor Bahru, Ministry of Health Malaysia Jalan Mahmoodiah, Johor Bahru, Johor. Malaysia.

**Keywords:** Pharmacists, Diabetes mellitus type 2, Medication adherence, Primary health care, Haemoglobin A glycosylated

## Abstract

**Introduction:**

The Diabetes Medication Therapy Adherence Clinic (DMTAC) programme is an ambulatory care service provided by trained pharmacists in collaboration with doctors. It supports improved glycaemic control by enhancing patients’ understanding and treatment adherence through education.

**Methods:**

Patients enrolled in the DMTAC programme in all 84 health clinics in Johor from January 2016 to December 2017 were included. Demographic characteristics, diabetic and medication history, current treatments, comorbidities and therapeutic outcomes (glycosylated haemoglobin A1c [HbAlc] level and lipid profile) were collected and reviewed retrospectively. Baseline HbAlc levels and lipid profiles were taken from patients’ medical records during their first visits and post-intervention values from follow-up data, including the most recent information documented during the last DMTAC pharmacist visit before December 2017.

**Results:**

A total of 1107 patients (693 women and 414 men) with type 2 diabetes mellitus aged 24–88 years were included. The mean HbA1c level significantly decreased by 0.92±1.94%. The mean total cholesterol (-0.27 mmol/L), low-density lipoprotein (-0.31 mmol/L) and triglyceride levels (-0.14 mmol/L) slightly but significantly decreased. Both systolic and diastolic blood pressures also showed small but significant reductions from 142/81 mmHg pre-intervention to 137/79 mmHg postintervention. The patients’ understanding scores improved from 87.94% to 98.55% after their fourth DMTAC pharmacist visit.

**Conclusion:**

The DMTAC programme in public health clinics across Johor, Malaysia, significantly improves HbA1c levels, fasting blood glucose levels, lipid profiles, BPs and medication understanding, with DMTAC pharmacists playing a key role in supporting multidisciplinary teams through targeted interventions and counselling.

## Introduction

Diabetes mellitus (DM) is a major global public health problem and is emerging as a pandemic. In 2021, approximately 537 million people worldwide had DM, with this number projected to rise to 643 million by 2030 and 783 million by 2045. Over 6.7 million people aged 20-79 years were estimated to have died from DM-related causes^1^

According to the International Diabetes Federation, Malaysia has one of the highest DM prevalence rates in Asia, with 20.0% of adults aged 20-79 years affected.^1^ The 2023 National Health and Morbidity Survey estimated that approximately 2,210,457 individuals in Malaysia had known DM, with most receiving treatment at Ministry of Health (MOH) health clinics (77.5%), followed by MOH hospitals (9.4%), private clinics (8.3%) and private hospitals (2.6%).^2^ DM is a life-long disease, and optimal glucose control can be achieved through strict adherence to medications and dietary guidelines, thereby minimising long-term complications. Collaborative management among healthcare professionals has been recognised as a key factor in improving glycaemic control and DM-related health outcomes.^3^ Pharmacists represent the third-largest healthcare profession in the world.^4^ As medication experts, they are uniquely positioned to collaborate with other healthcare professionals and assist in medication therapy management, which is crucial for managing complex diseases that often involve multiple comorbidities, such as type 2 diabetes mellitus (T2DM).^5^ Pharmacists’ responsibilities include ensuring the quality and evidence-based use of medications, providing adherence support and offering lifestyle education.^4^

Multiple studies or clinical trials have demonstrated that reducing the glycosylated haemoglobin A1c (HbAlc) level significantly lowers the risk of chronic microvascular and macrovascular complications associated with poor DM control.^6-8^ The Diabetes Control and Complications Trial (DCCT) demonstrated a relationship between an elevated HbAlc level and an increased risk of complications in type 1 diabetes mellitus,^8^ with similar findings for T2DM in the UK Prospective Diabetes Study (UKPDS).^7^ The DCCT suggested that achieving a glycaemic level as close to the nondiabetic range as safely possible can reduce the risk of retinopathy (76%), nephropathy (50%), neuropathy (60%) and cardiovascular disease (CVD) (4l%).^8^ The UKPDS further linked a 1% reduction in the HbAlc level to a 21% lower risk of any DM-related endpoint, a 21% reduction in DM-related deaths and a 37% decrease in microvascular complications.^7^

Despite advancements in DM management, several gaps persist in Malaysia, necessitating pharmacist intervention in primary healthcare clinics (PHCs). These gaps include poor medication adherence, suboptimal glycaemic control and challenges related to polypharmacy, particularly among older adult patients with comorbidities. Additionally, many patients lack personalised education on DM management, including medication use, self-monitoring of blood glucose, proper insulin injection technique and lifestyle modifications. There is also a need for improved management of DM-related complications, such as hypoglycaemia, and enhanced patient understanding of treatment regimens. Diabetes Medication Therapy Adherence Clinic (DMTAC) pharmacists can address these challenges by optimising medication therapy, providing tailored education, enhancing adherence and supporting patients in achieving better glycaemic control, ultimately improving health outcomes and preventing longterm complications.^9-12^

Pharmacists’ positive impact on DM management has been demonstrated in various settings, both in Malaysia and overseas. Pharmacist-led interventions have been associated with additional reductions in the HbA1c level across multiple healthcare settings, including hospitals,^9-11,13^ PHCs^5,12,14^ and communities.^15,16^ These reductions in the HbA1c level correlate with lower rates of CVDs and significant cost-savings for healthcare systems.^5^ Furthermore, pharmacist-led educational interventions have been shown to improve patients’ knowledge of their disease, its complications, treatment options (both pharmacological and non-pharmacological) and self-monitoring techniques.^4^ Primary care settings, such as ambulatory settings, outpatient clinics and healthcare centres, provide patients with convenient access to pharmacists, who serve as reliable healthcare providers willing to deliver direct medical care, particularly for T2DM management.^3^ The inclusion of pharmacists in DM care teams within primary care settings has been shown to reduce the HbA1c level, blood pressure (BP), hospitalisations and DM-related complications while improving medication adherence and patient quality of life and reducing mortality rates.^3,17,18^

However, most studies demonstrating positive outcomes of the DMTAC programme have been conducted in hospital settings, where patients often present with more comorbidities and complications and have access to a wider range of glucose-lowering drugs (GLDs) compared to PHCs. This difference in patient profile and medication availability may influence the outcomes of pharmacist-led interventions, making it essential to evaluate their impact specifically in primary care settings. Furthermore, while a few studies have explored the impact of the DMTAC programme in primary care settings, they have generally been limited in scale. For example, the only similar study involved just 14 health clinics within a single district,^12^ whereas our study comprehensively includes all 84 health clinics in Johor, making it the most extensive assessment of the DMTAC programme in primary care to date. By addressing these gaps, our study provides robust evidence on the effectiveness of pharmacist-led interventions in the primary care context, offering valuable insights that can inform policy and practice improvements within the Malaysian healthcare system.

As part of a multidisciplinary approach, the DMTAC programme is managed by trained pharmacists in collaboration with doctors, following the DMTAC protocol established by the Pharmaceutical Services Division of the MOH Malaysia. This initiative aims to enhance glycaemic control in patients with T2DM by improving their understanding of and adherence to treatment regimens through structured education.^9^ To further strengthen PHC services, the MOH Malaysia initiated the Enhanced Primary Healthcare programme in 2017, focusing on the detection and management of non-communicable diseases (NCDs) such as DM, hypertension and dyslipidaemia. As part of this initiative, the Medication Therapy Adherence Clinic (MTAC) concept has expanded to include other NCDs through the Cardiovascular Care Bundle-MTAC, which has been widely adopted at PHCs.^19^

The DMTAC programme was introduced in PHCs across Malaysia in 2011. It became operational in Johor in January 2012. During clinic visits, patients are enrolled in the DMTAC programme based on the following inclusion criteria as outlined in the protocol: (i) uncontrolled T2DM despite optimal medication and dosing, (ii) non-compliance with prescribed medications, (iii) HbA1c levels of >8.0%, (iv) presence of comorbidities or multiple medications or (v) microvascular or macrovascular complications.^6^ Patients are identified through referrals from doctors or pharmacists during routine follow-up visits. Upon enrolment, they are scheduled for follow-up appointments with pharmacists at intervals ranging from 1 to 3 months, with a minimum of four consecutive visits. During each visit, patients receive personalised counselling and education based on materials developed by the Pharmaceutical Services Division of the MOH. The educational content is delivered in four primary modules: an overview of DM and its treatment, management of comorbidities such as hypertension and hypercholesterolemia, recognition and management of hypo- and hyperglycaemia and adoption of healthier lifestyle choices (e.g. diet and exercise). Additionally, the prevention of microvascular and macrovascular complications associated with poor glycaemic control is discussed. Additional topics, such as foot care and sick-day management, are addressed as needed based on patients’ level of understanding. At each visit, DMTAC pharmacists assess medication adherence and patient understanding of their prescribed treatment regimen. During joint follow-up appointments with doctors, pharmacists may recommend medication adjustments, including the intensification or de-escalation of insulin therapy, oral glucose-lowering drugs (OGLDs), antihypertensive medications, statins or antiplatelet agents. All assessments and interventions conducted during DMTAC sessions are documented either electronically or manually in DMTAC notes.

The DMTAC programme empowers pharmacists to monitor glycaemic control between doctor visits, provide self-management education and develop individualised management plans for patients. This study aimed to assess the impact of pharmacist-led interventions within the DMTAC programme on diabetic and metabolic outcomes. By demonstrating the positive effects of the programme in managing T2DM, this study also sought to advocate for the expansion of MTACs to address other NCDs in PHCs across Malaysia.

## Methods

### Study design and settings

A retrospective multicentre study evaluating the effects of the DMTAC programme on the glycaemic and therapeutic outcomes of patients with T2DM was conducted in Johor, Malaysia. Patients enrolled in the DMTAC programme across 84 health clinics in Johor from January 2016 to December 2017 were included. Patients who did not have pre- and post-intervention HbA1c results and lipid profiles, had fewer than four follow-up DMTAC visits or had defaulted visits for more than 3 months were excluded from this study. The progress notes in each patient’s medical records (developed by the MOH Malaysia) and pharmacist-led interventions were reviewed retrospectively. Data such as patient demographics, diabetic and medication history, current treatments, comorbidities and therapeutic outcomes (HbA1c levels and lipid profiles) were recorded using a data collection form approved by the NMRR. The form was developed by the primary investigator based on clinical guidelines and existing literature and was tested and refined through a pilot study 20 conducted before this research to ensure clarity and relevance. Baseline HbA1c levels and lipid profiles were taken from patients’ medical records during their first visits, while post-intervention values were obtained from follow-up data, specifically from the most recent visit to DMTAC pharmacists before December 2017.

Based on a pilot study evaluating the outcomes of the DMTAC service at Mahmoodiah Health Clinic, Johor Bahru,^20^ a minimum sample size of 24 was required. This figure was determined using the Power and Sample Size Calculation software version 3.1.2, based on a significance level of 0.05, a power of 80% and an assumed mean HbA1c level difference of 1% with a standard deviation (SD) of 1.51.

### Study outcomes

The primary outcomes were changes in the HbA1c level (%) and fasting blood glucose (FBG) level, as well as lipid profile parameters, including the total cholesterol (TC) level, low-density lipoprotein (LDL) level, high-density lipoprotein (HDL) level and triglyceride (TG) level before and after DMTAC pharmacist involvement. The secondary outcomes included patients’ systolic blood pressure (SBP), diastolic blood pressure (DBP), body mass index (BMI) and medication understanding score (%). The medication understanding score was assessed based on patients’ knowledge of the correct dosage, frequency, indication and timing of each prescribed medication at every visit. A ‘tick’ was recorded for each correct answer in the assessment column. The percentage of medication knowledge was calculated as the number of ticks (correct responses) divided by the total possible number of ticks for all prescribed medications.

Patients’ medication regimens, including OGLDs and insulin therapy, were also reviewed. Treatment targets were considered acceptable when they aligned with the Clinical Practice Guideline (CPG) on the Management of T2DM^6^ published by the MOH Malaysia.

### Statistical analysis

Data were coded and entered into SPSS software version 24.0 (IBM Corp., Armonk, New York, United States) for statistical analysis.

Demographic data were analysed using descriptive statistics. For continuous variables, normality was assessed through frequency distribution (histogram). Paired t-tests were used to analyse differences between the pre- and post-intervention HbA1c levels (%), FBG levels (mmol/L), TC levels (mmol/L), LDL levels (mmol/L), HDL levels (mmol/L), TG levels (mmol/L), SBP (mmHg), DBP (mmHg), BMI (kg/m^2^) and medication understanding scores (%), as these data were normally distributed and were represented as mean values. The results were considered statistically significant at a P-value of <0.05.

## Results

This study included 84 government-operated PHCs, with a total of 1287 newly recruited DMTAC patients from January 2016 to December 2017. Among them, 1107 patients (693 women and 414 men) with T2DM, aged 24-88 years, were eligible for inclusion. A total of 886 (80.0%) had been diagnosed with T2DM for more than 5 years, and over half (57.7%) were classified as obese, with a BMI of ≥27.5 kg/m^2^. The demographic characteristics and comorbidities are summarised in Table 1. Regarding DM complications, CVDs (e.g. coronary heart disease, stroke and peripheral vascular disease) were the most common complication among the patients (45.1%), followed by nephropathy (31.5%). All patients were treated with either OGLDs (23.4%), insulin only (15.7%) or insulin combined with OGLDs (60.9%) during study recruitment, as presented in Table 2.

**Table 1:** Demographic characteristics and comorbidities of the patients.

Characteristics	No. of patients (%) (N=1107)
Age (year), mean (SD) 57.99 (9.93)	
**Sex**	
Male	414 (37.4%)
Female	693 (62.6%)
**Ethnicity**	
Malay	855 (77.2%)
Chinese	192 (17.3%)
Indian	57 (5.2%)
Others	3 (0.3%)
**Marital status**	
Single	35 (3.2%)
Married	1072 (96.8%)
**Smoker**	
Yes	93 (8.4%)
No	1014 (91.6%)
**Alcohol intake**	
Yes	32 (2.9%)
No	1075 (97.1%)
**Number of years diagnosed with type 2 diabetes mellitus**	
<5	221 (20.0%)
5-10	488 (44.1%)
10-20	330 (29.8%)
>20	68 (6.1%)
**Body mass index (kg/m2)**	
<18.5 (Underweight)	132 (11.9%)
18.5-22.9 (Normal)	336 (30.4%)
23.0-27.4 (Pre-obesity)	504 (45.5%)
27.5-32.4 (Obesity I)	85 (7.7%)
32.5-37.4 (Obesity II)	37 (3.3%)
>37.5 (Obesity III)	13 (1.2%)
Comorbidities	n=590
Cardiovascular	266 (45.1%)
Nephropathy	186 (31.5%)
Neuropathy	40 (6.8%)
Retinopathy	89 (15.1%)
Foot	9 (1.5%)

**Table 2:** Glucose-lowering drug regimen.

Type of glucose-lowering medication regimen	Number of patients (N=1107)		n	%
OGLDs only	259 (23.4%)	Monotherapy	47	4.2
		Dual therapy	202	18.3
		Triple therapy	10	0.9
OGLDs with insulin	674 (60.9%)	OGLDs + premixed insulin	432	39.0
		OGLDs + basal insulin or OGLDs + basal and bolus insulin regimen	242	21.9
Insulin only	174 (15.7%)	Basal insulin	2	0.2
		Premixed insulin	122	11.0
		Basal and bolus insulin regimen	50	4.5

OGLD: oral glucose-lowering drug

For the primary outcomes, a significant difference in the mean HbAlc level was observed between baseline and follow-up, decreasing from 10.3% (89 mmol/mol) to 9.4% (79 mmol/ mol), resulting in a mean reduction of 0.9% (Table 3). Among 849 patients who had HbAlc level reductions after the DMTAC programme, 529 (62.3%) had a reduction of more than 1%. The FBG level significantly decreased from 10.89 mmol/L to 8.69 mmol/L, with a mean reduction of 2.19 mmol/L. For the lipid profiles, a slight but significant reduction was observed in the TC level (–0.27 mmol/L), LDL level (–0.31 mmol/L) and TG level (–0.14 mmol/L). The HDL level slightly increased from 1.19 mmol/L to 1.21 mmol/L (+0.2 mmol/L), while the BMI slightly increased by +0.05 kg/m^2^, although both results were statistically insignificant. Both SBP and DBP showed small but significant reductions, decreasing from 142/81 mmHg to 137/79 mmHg. The patients demonstrated improved medication understanding scores after their fourth visit to DMTAC pharmacists, increasing from 87.94% to 98.55%.

**Table 3:** Patient outcomes pre- and post-DMTAC intervention.

Outcome measure	Mean (SD)		Mean difference (SD)	P-value
	Pre-intervention	Post-intervention		
**Primary outcomes**				
HbA1c level (%)	10.3 (2.01)	9.4 (2.04)	-0.9 (1.94)	0.000
FBG level (mmol/L)	10.89 (3.69)	8.69 (4.96)	-2.20 (5.37)	0.000
TC level (mmol/L)	5.42 (1.29)	5.15 (1.65)	-0.27 (1.50)	0.000
LDL level (mmol/L)	3.31 (1.16)	2.99 (1.11)	-0.32 (0.99)	0.000
HDL level (mmol/L)	1.27 (0.37)	1.29 (0.36)	+0.02 (0.02)	0.066
TG level (mmol/L)	2.02 (1.22)	1.88 (1.18)	-0.14 (0.99)	0.000
**Secondary outcomes**				
Systolic BP (mmHg)	142.57 (41.79)	137.46 (24.23)	-5.11 (46.30)	0.000
Diastolic BP (mmHg)	81.14 (10.04)	79.28 (9.72)	-1.86 (10.68)	0.000
Understanding score (%)	87.94 (19.15)	98.55 (5.07)	+ 10.61 (18.13)	0.000
BMI (kg/m^2^)	28.71 (5.28)	28.76 (5.85)	+0.06 (3.28)	0.566

## Discussion

Our results suggest that DMTAC services in primary care settings significantly improve the HbA1c and FBG levels. The findings align with previous reports demonstrating a positive association between pharmacist-led interventions and improved glycaemic control.^5,9-13^ The HbA1c level reduction of 0.9% (SD=1.94) and FBG level reduction of 2.2 mmol/L (SD=5.37) in our study were statistically significant and are comparable to findings from systematic reviews and meta-analyses.^18,21^ Notably, 62.3% of the patients in this study achieved at least a 1% reduction in their HbA1c level after participating in the DMTAC programme. The significant improvement in the FBG level observed in this study is consistent with the findings of Al Mazroui *et al.^22^* and the Fremantle Diabetes Study.^23^ Pharmacists play a crucial role in educating patients about insulin requirements and addressing barriers to insulin initiation, such as fear of needles and concerns about hypoglycaemia. This role may explain the improvement in the HbA1c and FBG levels observed in our population.

Although reductions in the HbA1c level are essential for reducing DM-related complications, intensive glycaemic control alone has a limited impact on reducing CVD risks in patients with T2DM, except in overweight patients treated with metformin.^24^ Other major CVD risk factors, including hypertension, hyperlipidaemia and obesity, must also be effectively managed. High baseline LDL and TC levels are well-established risk factors for CVD, whereas HDL is considered protective due to its inverse association with atherosclerotic risks.

Patients with T2DM are particularly vulnerable to the harmful effects of increased LDL levels due to the presence of small dense LDL particles and the oxidation of glycated LDL.^25^ In this study, a significant mean reduction in the LDL level of -0.32 mmol/L was observed, bringing the average LDL level to 2.99 mmol/L. Notably, 40.9% of the patients achieved the target LDL level of <2.6 mmol/L, as recommended by the Malaysian CPG, compared to only 26.6% before joining the DMTAC programme. Among 67.8% of the patients who experienced LDL level improvement, 30.9% had a reduction of more than 1 mmol/L. A slight increase of 0.02 mmol/L in the HDL level was also observed, although it was not statistically significant. However, more than half of the patients (53.7%) showed an improvement in their HDL level, with 65.5% achieving an increment of more than 0.1 mmol/L. Additionally, a slight but significant reduction of -0.14 mmol/L in the TG level was noted, with 53.3% of the patients attaining the target TG level of <1.7 mmol/L.^6^ Previous studies have indicated that each 1-mmol/L increase in the LDL level is associated with a 1.57-fold higher risk of CVD, whereas a 1-mmol/L reduction corresponds to a 36% risk reduction.^26^ Moreover, patients with LDL levels above 3.9 mmol/L are 2.3 times more likely to develop CVD than those with LDL levels below 3 mmol/L. Despite LDL level control with statins, elevated TG levels remain an independent risk factor for CVD in patients with DM.^27^

The modest improvements observed in the lipid profiles of the patients in this study may be attributed to several factors, including the limited selection of lipid-lowering agents available at the PHCs, the duration of antihyperlipidemic therapy and the frequency of lipid level assessments. During the study period, simvastatin was the only statin that medical officers could prescribe and initiate at the governmental PHCs. Atorvastatin required consultation with a family medicine specialist, while other statins were unavailable at the PHCs. Simvastatin was typically prescribed at doses of 20 mg or 40 mg, categorised as medium-intensity statin therapy. Although 80 mg simvastatin has been shown to lower LDL levels by an additional 6% over the 40 mg dose, the FDA advises against increasing the dose to 80 mg due to the risk of muscle injury.^28^ Furthermore, the Malaysian CPG recommends lipid level monitoring every 3 months during antihyperlipidemic therapy intensification. However, most patients with T2DM at governmental PHCs undergo lipid tests only annually, with some experiencing additional delays due to high patient loads. Given that 59.1% of the patients in this study still had LDL levels above 2.6 mmol/L, more aggressive lipid-lowering strategies are necessary to reduce overall CVD risks in patients with T2DM. These strategies may include increasing access to high-intensity statins, optimising therapy regimens and improving the frequency of lipid monitoring in PHCs.

Apart from pharmacological interventions, lifestyle modifications, including dietary changes and physical activity, play a critical role in improving lipid profiles. A diet rich in unsaturated fats, fibre and plant sterols while reducing intake of saturated fats, trans fats and refined carbohydrates has been shown to lower LDL and TG levels while increasing HDL levels.^29^ Regular physical activity, particularly aerobic exercises such as brisk walking, cycling and swimming, is associated with improved lipid metabolism and can help lower LDL levels while raising HDL levels.^30^ However, adherence to dietary recommendations and exercise regimens remains a challenge for many patients, particularly those with limited access to structured lifestyle intervention programmes. Addressing these lifestyle factors through patient education, counselling and support programmes could enhance lipid profile improvements and reduce CVD risks in patients with T2DM.^30,31^

Our study demonstrated a significant reduction of BP of 5.1/1.86 mmHg, from 142/81 mmHg to 137/79 mmHg, which is close to the target BP for patients with T2DM according to the Malaysian CPG.^6^ Hypertension is a major risk factor for both CVD and microvascular complications. The optimal BP for patients with T2DM is still uncertain. The recent Malaysian CPG has advised a treatment goal of lower than 139 mmHg for SBP and lower than 79 mmHg for DBP.^6^ The American Diabetes Association recommends an SBP/DBP on-treatment target goal of <130/80 mmHg if it can be safely attained, consistent with guidelines from the American College of Cardiology and the American Heart Association.^32^ Despite discrepancies in recommendations, patients with T2DM should be treated to achieve a target BP of at least <140/80 mmHg to reduce CVD and microvascular complications. A previous metaanalysis has suggested that achieving an SBP target of 138 mmHg reduces all-cause mortality, coronary heart disease, heart failure and stroke in patients with DM.^33^

Given the complexity of current medication therapies and the emphasis on patient medication safety, the need for pharmacists to improve patient knowledge, understanding and adherence to medication regimen has become more evident. Good medication knowledge is essential for effective DM management. In our study, the patients’ medication understanding scores improved significantly from 87.9% to 98.6% after four visits to DMTAC pharmacists, consistent with several local findings.^12,34^

Although the increase in the BMI observed at the end of the study was modest and statistically insignificant, 57.8% of the patients were either overweight or obese, with a mean BMI of 28.7 kg/m^2^. Obesity is closely linked to insulin resistance, a key factor in the pathogenesis of T2DM, and together, they increase an individual’s mortality risk sevenfold.^35^ The Look AHEAD Study demonstrated the benefits of weight loss in patients with T2DM, showing that a 5%-10% reduction in body weight can improve overall fitness, lower HbA1c levels, reduce CVD risks and decrease the need for GLDs, antihypertensives and lipid-lowering medications after 1 year.^35^ Approximately 76.6% of the patients in this study were on insulin or dual combination therapy (insulin with OGLDs). The modest weight gain observed may partly be attributed to increased insulin doses administered during DMTAC follow-up visits. Additionally, OGLDs can be broadly categorised into those associated with weight gain and those that are either weight-neutral or promote weight loss. Apart from metformin, which is typically used as a first-line OGLD, sulfonylureas (e.g. gliclazide or glibenclamide) are among the most commonly prescribed OGLDs in government PHCs, despite their potential to promote weight gain. DMTAC pharmacists play a crucial role in addressing this issue by advising doctors on treatment options with a lower risk of weight gain, such as metformin and dipeptidyl peptidase-4 inhibitors. They also educate patients about the potential weight gain associated with insulin and appetite-stimulating OGLDs, such as sulfonylureas. During the study period, newer agents such as sodium-glucose cotransporter-2 inhibitors and insulin analogues were not yet available at the PHCs. These agents could have been recommended for obese patients with poorly controlled T2DM to support both weight management and glycaemic control.

Despite the significant improvements observed in the HbA1c level, BP and lipid profile, the inherent limitations of this study were acknowledged, particularly concerning potential confounding factors. Given the pre-post-interventional study design, the observed changes in the clinical outcomes may have been influenced by various factors beyond pharmacist-led interventions. For instance, patients might have been initiated on new GLDs (including insulin), adjusted their antihypertensive or lipid-lowering therapy dose or received additional counselling from dietitians and physiotherapists during the study period. Variations in lifestyle modifications, adherence levels and intrinsic disease progression could also contribute to the observed changes. Additionally, differences in the pre-post duration among individual patients may have introduced variability in the measured outcomes. While DMTAC pharmacists play a crucial role in ensuring medication adherence, providing patient education and identifying pharmaceutical care issues, the multifactorial nature of DM management necessitates a holistic approach involving multiple healthcare providers.

Nevertheless, the DMTAC programme provides a structured and sustained pharmacist-led intervention, which may act as a key facilitator in optimising therapy adherence and medication management. Pharmacists reinforce adherence to prescribed regimens; address medication-related concerns such as medication side effects, insulin adherence barriers, insulin injection technique and drug-drug interactions; and mitigate hypoglycaemia fears. DMTAC pharmacists also collaborate with doctors to ensure timely treatment modifications. These interventions may have contributed to the favourable clinical outcomes observed in our study, despite the presence of confounding variables. While the study design does not allow us to establish causality, the findings align with previous literature demonstrating the beneficial impact of pharmacist-led interventions in DM management. The significant improvements observed in the HbA1c level, BP and lipid profile suggest that structured DMTAC pharmacist-led interventions may enhance the effectiveness of standard DM care by reinforcing adherence, optimising therapy and ensuring timely intervention. To further strengthen the evidence, future studies employing a randomised controlled trial design or including a control group receiving usual care should be conducted to help minimise the influence of confounders and better isolate the impact of pharmacist-led interventions. Additionally, collecting detailed data on lifestyle modifications, dietitian and physiotherapist involvement and medication adjustments would provide a clearer understanding of the independent contribution of pharmacist-led interventions.

### Limitations

This study has several limitations. Given the pre-post-interventional and retrospective study design, the outcomes may have been influenced by confounding factors such as medication changes, additional counselling and lifestyle variations. Furthermore, missing or inconsistent data presented challenges, and the follow-up duration for some patients was less than a year, limiting conclusions on the long-term efficacy of DMTAC services. The absence of a control group further restricts comparisons between DMTAC interventions and standard care. Longer follow-up periods and controlled studies are necessary to confirm the sustained benefits of DMTAC services.

## Conclusion

The DMTAC programme at PHCs in Johor, Malaysia, significantly improves the HbA1c level, FBG level, lipid profile, BP and medication understanding of patients, with DMTAC pharmacists playing a crucial role in supporting multidisciplinary teams through targeted interventions and counselling.

## Data sharing statement

The patients’ names were recorded with a study identification number in this research. Instead of patient identifiers, code numbers were used on the subject data sheets. All data were entered into a computer. Upon completion of the study, the data on the computer were copied to a pen drive, and the data on the computer were then erased. The pen drive and any hard copy data will be stored in a locked office of the investigators and maintained for a minimum of 3 years after the completion of the study. After this period, the data on the pen drive and any hard copies will be destroyed. The patients will not be able to view their study data, which will be consolidated into a database.

**How does this paper make a difference in general practice?**

- Pharmacist-led interventions in the Diabetes Medication Therapy Adherence Clinic (DMTAC) programme in primary care settings improve patients’ diabetic and metabolic outcomes. Therefore, the Medication Therapy Adherence Clinic concept can be expanded to manage other non-communicable diseases across primary healthcare clinics (PHCs) in Malaysia.
- Given the shortage of registered diabetes educators in PHCs, trained DMTAC pharmacists play an important role in supporting comprehensive care delivery. As integral members of multidisciplinary healthcare teams, DMTAC pharmacists can help address gaps in care by providing essential support in diabetes mellitus management, complementing the valuable contributions of diabetes educators and other healthcare professionals.
